# Exploring current cancer screening practices and guidelines for adults under age 50 in the United States

**DOI:** 10.3389/fpubh.2026.1816070

**Published:** 2026-06-08

**Authors:** Jamie M. Reedy, Nancy A. Borstelmann, Veda N. Giri

**Affiliations:** 1 Early Onset Cancer Program, Yale Cancer Center, Yale School of Medicine, New Haven, CT, United States; 2 Yale Child Study Center, Yale School of Medicine, New Haven, CT, United States

**Keywords:** cancer risk, cancer screening, early detection, early onset cancer, patient-centered care

## Abstract

The concerning rise in early onset cancer (EOCs), defined as cancers diagnosed under the age of 50, has called attention to identifying individuals who may benefit from earlier cancer screening. These approaches involve patient-centered discussions to establish a personalized, risk-based screening plan. Important factors to consider when establishing a personalized risk-based cancer screening plan include a comprehensive family and personal cancer history and, if appropriate, genetic testing. Limitations in current cancer screening initiatives, healthcare systems, provider and public awareness, and access issues constrain these efforts. Within this context, we explore the current landscape of cancer screening for younger adults in the United States, describe associated challenges and efforts, and propose strategies for risk-based cancer screening in young adults.

## 1 Introduction

There has been a concerning 79.1% global rise in incidence between 1990 and 2019 of early onset cancer (EOC), defined as cancers diagnosed under age 50 (1–5). EOC diagnoses now account for 12% of all cancer diagnoses (6). The largest increased incidence of EOCs has been noted in breast, colorectal, kidney, and uterine cancers, with early onset colorectal cancer rates rising the fastest (2, 4). Women under age 50 represent 67% of all EOC diagnoses, attributed primarily to early onset breast cancer (7, 8). Early onset colorectal cancer (CRC) has been rapidly rising and is often diagnosed at advanced stages (9, 10). Despite the general decline of overall cancer mortality over the last 20 years (6, 11), EOC mortality rates have risen 27.7%, driven by increased mortality in colorectal, uterine, and testicular cancers (2, 3, 12). The drivers of rising EOC incidence and mortality rates are likely multifactorial (e.g., exposome changes, microbiome disruptions, lifestyle and environmental exposures, comorbidities), including genomic and hereditary factors (1, 7, 12–14). Approximately 10–20% of EOCs are attributable to germline pathogenic variants (inherited mutations in cancer genes), with higher estimates observed in certain tumor types such as colorectal cancer (15–17). Many EOCs also display more aggressive biology and worse clinical outcomes compared to average and later onset cancer diagnoses (12). More research on the etiology and influencing factors of early onset cancers is still needed.

With heightened attention on EOC, the need to increase awareness and uptake of risk-based cancer screening under age 50 has become a critical focus for both the professional community and the public. While cancer screening has been shown to save lives (18), significant limitations and barriers to these efforts remain (6). Substantial disparities in cancer screening exist across racial, geographic, sociodemographic, and healthcare access factors (19–21). Hispanic/Latine, Black, and Asian individuals are less likely than White individuals to receive a physician recommendation for cancer screenings or genetic testing (20). This discrepancy is particularly concerning given that earlier screening for CRC can lead to an estimated 19% reduction in mortality among Black individuals (22). Furthermore, while evidence supports efforts at early detection, most screening guidelines are largely determined at the population level and based primarily on average risk and age (23). Consequently, individuals with a potential risk of EOC may lack a clear pathway to risk assessment and early detection.

To address these gaps, public health campaigns and national screening programs are essential strategies in cancer control. These efforts include raising awareness of specific cancer risk factors and potential signs and symptoms, dispelling misconceptions, and promoting equitable access to screening services (24). Barriers to access are critical factors to determine at the level of each community and include sociocultural factors such as health literacy, economic challenges, patient beliefs, and mistrust of health care among minoritized individuals, among others (25). Such initiatives are also enhanced through emphasis on the importance of considering family cancer history as a critical component in risk assessment for younger individuals not yet eligible for standard age-based screening.

Moving forward, further research is needed to better identify individuals at risk for EOC and determine how to screen effectively while minimizing risks and managing positive screening results (26, 27). The objective of this commentary is to examine the current landscape of cancer screening among younger adults in the United States, delineate the associated challenges, highlight ongoing public health efforts, and propose strategies to advance risk-based screening approaches in this population.

## 2 Screening overview

### 2.1 Screening guidelines

Current screening guidelines in the US are typically based on average cancer risk, balancing the benefits against risks at the population level. Screening for individuals under age 50 is usually recommended based on cancer risk factors, which include family cancer history, known genetic mutations indicating hereditary cancer syndromes, or specific exposures such as radiation. National guidelines exhibit variability in screening initiation and the factors that may influence these decisions (Table 1). In general, the National Comprehensive Cancer Network (NCCN) provides comprehensive family history- and gene-specific guidance on screening strategies for high-risk individuals (28). The American Cancer Society (ACS) supports early screening in specific scenarios and often defers details of screening to genetics professionals or healthcare specialists (29). The Centers for Disease Control and Prevention (CDC) upholds the guidelines from the US Preventive Services Task Force (USPSTF) (23, 30), which generally focus more on population-level cancer screening and less on high-risk individuals. Table 1 highlights key aspects of early cancer screening recommendations from various professional organizations (23, 28–33).

**Table 1:** High-risk cancer screening guidelines for young individuals^*^.

High-risk group	NCCN	ACS	USPSTF/CDC
Colorectal cancer			
Lynch Syndrome (*MLH1, MSH2, MSH6, PMS2, EPCAM*)	For *MLH1, MSH2*, and *EPCAM*: colonoscopy starting age 20–25, every 1–2 years For *MSH6 and PMS2*: colonoscopy starting age 30–35, every 1–3 years	Early & frequent colonoscopy recommended; defers to genetics	Excluded
FAP/MAP (*APC, MUTYH* biallelic)	Start age 10–12, colonoscopy or sigmoidoscopy every 1–2 years	Recognized as extremely high risk	Excluded
1st-degree relative with CRC < 60	Colonoscopy age 40 or 10 years earlier, every 5 years	Same as NCCN	Excluded
**IBD (ulcerative colitis or Crohn's colitis)**	Begin 8–10 years after diagnosis, every 1–3 years	Acknowledges increased risk	Excluded
Breast cancer			
*BRCA1/BRCA2* mutation	Breast MRI age 25, mammogram age 30, annual both	Annual MRI + mammogram starting ~age 30	Excluded
Other genes (*PALB2, TP53, PTEN, CDH1, STK11*)	Gene-specific early MRI ± mammogram	Recognizes high risk; specialist-guided	Excluded
Chest radiation < 30 yrs	MRI + mammogram age 25 or 8 yrs post-radiation, annual	Same as NCCN	Excluded
Strong family history (≥20% lifetime risk)	MRI + mammogram based on risk models	MRI recommended if ≥20–25% lifetime risk	Excluded
Cervical cancer			
HIV/immunocompromised	Begin earlier; annual Pap/HPV	Same as NCCN	Excluded
DES exposure in utero	Annual Pap + vaginal cytology for life	Same as NCCN	Excluded
History of CIN2+	Continue screening ≥25 years after treatment	Same as NCCN	Acknowledged, but specifics deferred
Prostate cancer			
*BRCA2* mutation	PSA discussion starting age 40; consider annual testing	Discussion age 40–45	Not addressed
African American individuals	Earlier shared decision-making	Discussion starting age 45	Evidence insufficient for tailored guidance
Strong family history	PSA discussion age 40–45	Same as NCCN	USPSTF guidance limited to ages 55–69

^*^These guidelines are relevant for individuals ≤ 45. NCCN, National Comprehensive Cancer Network; ACS, American Cancer Society; USPSTF, US Preventive Services Task Force; CDC, Centers for Disease Control and Prevention; FAP, Familial Adenomatosis Polyposis; MAP, MUTYH-associated polyposis; CRC, Colorectal Cancer; IBD, Inflammatory Bowel Disease; CIN2±, Cervical Intraepithelial Neoplasia Grade 2 or higher.

The most informative family cancer history includes all cancers in relatives on both maternal and paternal sides of the family, age of cancer diagnoses in relatives, degree of relatedness to the individual, screening or prevention undergone by relatives, and if relatives passed away from cancers. All of this information may not be available to patients; therefore, when discussing cancer screening strategies, it may be necessary to rely only on the available family history. A comprehensive family history can guide clinical decisions about what age to begin specific cancer screenings (Table 1) (23, 28–30). For some cancers, such as colon cancer, family history alone can help inform age to begin colonoscopy. Current NCCN guidelines state that colorectal cancer screening should begin at age 40 or 10 years prior to the age of colorectal cancer diagnosis in a first-degree relative (34).

Family history can also be crucial to considering hereditary cancer genetic testing to identify hereditary cancer syndromes (28). Key indications for genetic testing include strong family history of cancers, young age at cancer diagnosis, specific cancer pathologic or molecular subtypes, and precision therapy eligibility (28). Hereditary cancer syndromes can be important in guiding the age at which to begin cancer screening. For breast cancer screening, breast MRI may be recommended to start at age 25 and mammograms at age 30, and then annually for females with *BRCA* mutations or a history of chest wall radiation (35). For prostate cancer screening, a discussion of prostate-specific antigen (PSA) testing pros and cons is recommended at age 40–45 for males with *BRCA2* mutations or a strong family history of prostate cancer (28). Clinical breast exams are also recommended to begin at age 35 for male *BRCA2* carriers (24) (Table 1). For colorectal cancer, colonoscopies can be recommended to start at significantly younger ages, such as in a person's early 20s, and on an annual basis, particularly if individuals have genetic mutations linked with hereditary cancer syndromes such as Lynch syndrome or Familial Adenomatosis Polyposis (28).

Additional risk factors may influence the age at which to begin cancer screening (Table 1) (28). For example, females who have had chest wall radiation before age 30 should consider screening for breast cancer with MRI and mammogram starting at age 25, or 8 yrs after radiation, and then annually (35). Cervical cancer screening is recommended to begin earlier for females with HIV, DES exposure, or cervical intraepithelial neoplasia Grade 2 or higher (28, 36).

### 2.2 Screening rates in young adults

Recommendations by professional organizations on age to initiate cancer screening for average-risk individuals are aligned for some cancers and vary for others. The National Comprehensive Cancer Network (NCCN), American Cancer Society (ACS), and the US Preventive Services Task Force (USPSTF) all recommend starting cancer screening at these ages: breast cancer at age 40, colorectal cancer at age 45, and lung cancer at age 50 (for specific patients) (23, 29, 35). For cervical cancer screening, NCCN and USPSTF endorse starting at age 21, while ACS advocates starting at age 25 (23, 29, 35). For prostate cancer screening, recommendations include shared decision-making starting at age 45 (NCCN), 50 (ACS), or 55 (USPSTF) (23, 29, 35).

Uptake of population-level screening rates in the US has typically been collected through self-report surveys, such as the publicly available Behavioral Risk Factor Surveillance System Survey (BRFSS) (37). According to the most recent BRFSS and National Health Interview Survey (NHIS) data, 63% of US adults reported receiving the recommended age-based screening for breast, colorectal, or cervical cancer in 2022, rising to 67.4% in 2023 (38, 39). As of 2023, the NHIS reported that women ages 40–49 were 22% less likely to have received recommended breast cancer screening, and adults ages 45–49 were nearly 50% less likely to be up to date on recommended CRC screening (39). Other factors, such as Hispanic/Latino ethnicity, mixed-race status, low income, and US federal or state-funded low-cost insurance coverage, are associated with substantially lower screening rates (37–43). At the population level, there is limited data on the screening rates and behaviors of adults under the age of US screening guidelines, particularly those at high risk for early onset cancer (37).

One study reported that women with a first-degree relative diagnosed with breast cancer under age 50 are at a higher risk of developing early onset breast cancer (44); however, according to the NHIS only 31.6% of young women (aged 18+) with a family history of breast or ovarian cancer in the US reported having a discussion on genetic testing or early screening recommendations with a healthcare provider in 2023 (45). An even lower percentage (21.7%) of young adults (aged 18+) with a family or personal history of colorectal cancer reported having such key discussions with a provider (45). These findings point to the need to enhance clinical guidelines to prompt providers to initiate discussions with their patients about family cancer history to guide personalized risk-based genetic testing or screening recommendations. Patient-empowerment strategies are also needed to support patients in these risk-based screening discussions.

Global cancer screening rates vary widely due to factors such as differences in screening guidelines, the absence of or under-resourced national screening programs, limited or developing cancer control policies, limited data collection or reporting, COVID-19 pandemic impacts, and sociocultural factors impacting healthcare engagement (46–50). Due to the limitations in data reporting across regions and countries, comparisons can be challenging, particularly for adults ages 18–45 at risk for early onset cancer. To address these challenges, the Cancer Screening in Five Continents (CanScreen5) is a global data repository and large-scale initiative to monitor, evaluate, and improve cancer screening programs internationally. While efforts to improve cancer screening globally are critical, a significant gap remains in data on younger adults.

Interpretation of rising rates of EOC can vary. Some researchers view the rise as partly reflecting increased diagnostic testing, with only a small proportion of diagnoses resulting in clinically meaningful disease (46). Others have suggested that enhanced cancer surveillance stemming from genetic testing could be contributing to higher EOC incidence rates over time (1, 2, 6). However, a major concern is that rates of EOC in individuals in their 20s and 30s have increased, which is younger than the age to start screening for breast cancer (age 40) and screening for colon cancer (age 45) in the US. As such, further research and public campaigns are needed to raise awareness of how to assess an individual's risk for EOC and to promote adherence to screening guidelines.

### 2.3 Public health campaigns & national screening programs

Given the rising incidence of EOC over the last few decades, public health campaigns and advocacy efforts are beginning to tailor education to younger individuals who may be at higher-than-average risk or have a family history of cancer. US screening programs and health communication campaigns aim to educate the public by disseminating information through a combination of traditional and digital approaches, along with interactive tools such as risk assessments, to reach underserved populations, including those at risk of early onset cancer. Global efforts like CanScreen5 aim to support cancer screening programs and policies tailored to distinct countries and regions worldwide (47).

Notably, in the US, the CDC launched *Bring Your Brave* in 2015 with a primary focus on women aged 18–45, leveraging technology to improve access to information about breast cancer for younger women (48). The campaign's resources, updated in 2024, include video testimonials of young women living with breast cancer that address risk, family history, lifestyle, and survivorship. This campaign also initiated *My Motivated Moment*, a podcast series promoting action to learn about risk and enhance awareness among healthcare providers of risk factors for early onset breast cancer.

The Colorectal Cancer Alliance, along with other disease-specific organizations, focuses on education and outreach for younger individuals. The ACS and the National Colorectal Cancer Roundtable (NCCRT) work in strategic partnership to catalyze efforts on early onset colorectal cancer, early detection, family history, and genetic testing, including initiatives to activate and advance CRC screening in rural communities (49). The ACS has recommended routine human papillomavirus (HPV) testing and Pap smears for cervical cancer screening among young adults, and has partnered extensively with stakeholder organizations to increase HPV vaccination rates (29, 50). The CDC also delivers cancer prevention awareness campaigns on HPV vaccination (“*HPV vaccine is cancer prevention”)* and healthy lifestyle promotion (30). These concerted efforts to vaccinate youth have been highly successful, consistently reporting reductions in the development of cervical cancer; for those vaccinated at or before age 16, risk reduction was 80% (51).

While few campaigns are tailored specifically to younger adults, some organizations and initiatives are addressing awareness of family cancer history. The CDC offers a multilingual online tool, *My Family Health Portrait*, that provides a structure for organizing family health history, including cancer, which can be shared with healthcare providers (52). Monthly awareness campaigns (e.g., October - breast cancer, March - colorectal cancer) further support these efforts by raising community awareness of cancer risk and promoting screening (49). Organizations like Facing Our Risk of Cancer Empowered (FORCE) provide tailored support for individuals at increased genetic risk of cancer, including guidance related to genetic testing and enhanced cancer prevention (e.g., prophylactic surgeries) (53). Other professional organizations, such as the National Society of Genetic Counselors (NSGC), also educate the public about the importance of family history and genetic counseling for early detection and prevention.

Initiatives have also begun to emphasize education for primary care and other medical audiences about personalized risk-based screening (PRBS), genetic testing, symptom recognition, and recommended diagnostics among younger individuals (54). The ACS Risk Assessment and Screening Toolkit offers professional education materials to primary care providers to promote early detection of familial, hereditary, and EOCRC, including information on specific diagnostic testing for younger patients (49).

## 3 Discussion

**Figure 1:**
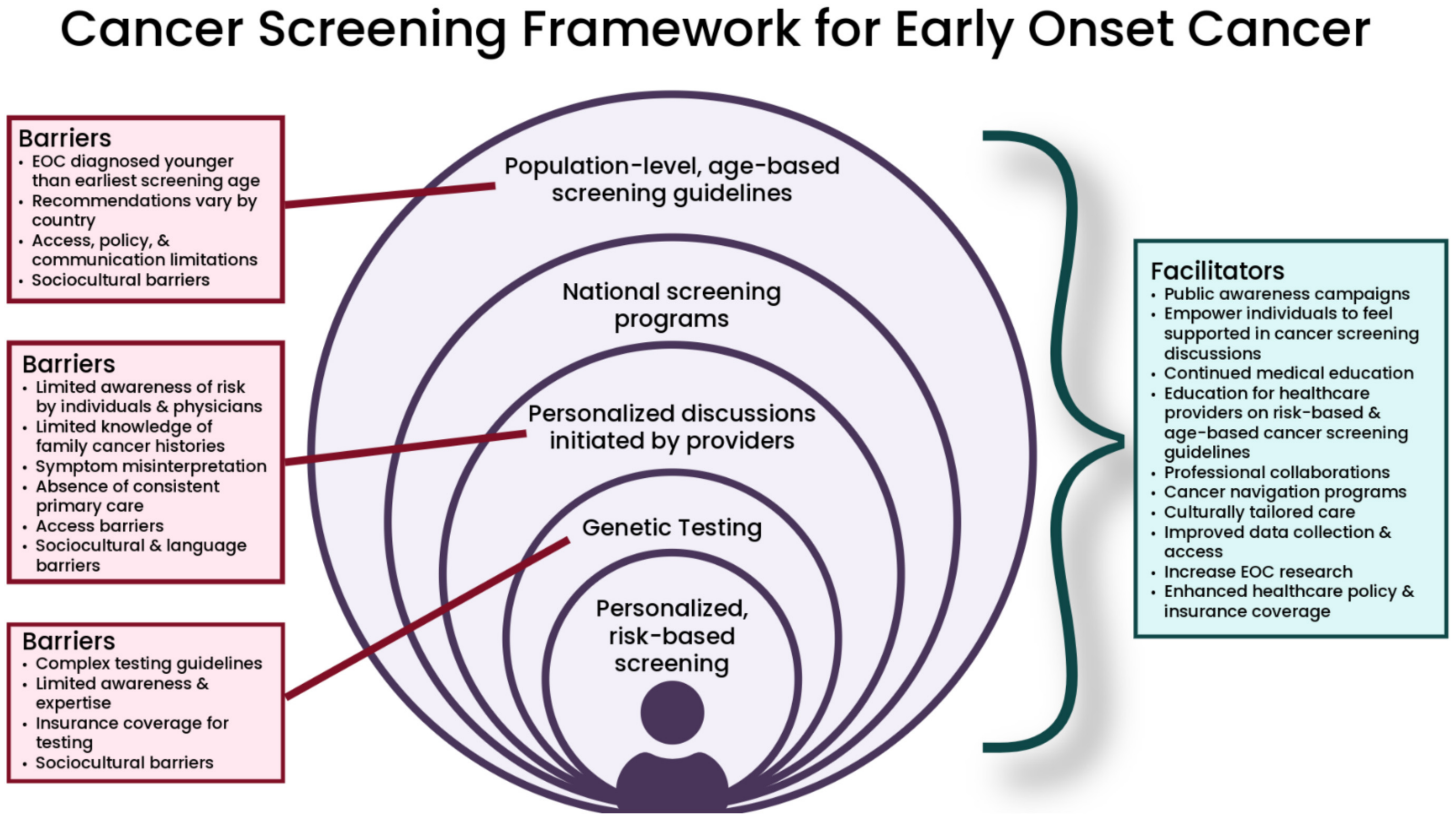
Cancer screening framework for early onset cancers. This figure highlights the multilevel US cancer screening guidelines with intersecting barriers, demonstrating the challenges associated with effective screening among adults younger than the earliest age of cancer screening guidelines. Facilitators across levels are also indicated.

With the concerning rise in EOC incidence and mortality rates, early cancer detection in younger adults is essential for improving outcomes. However, public and health professional awareness of risk, early warning signs and symptoms, and risk-based cancer screening guidelines are limited (55, 56). Additionally, complex and intersecting barriers complicate implementation at each level of potential screening intervention (see Figure 1). Thus, a critical need exists for enhanced awareness of EOC risk, symptom identification, and PRBS guidelines (57). Several strategies for action across each level of the socioecological model to facilitate enhanced personalized, risk-based cancer screening guidelines for adults under age 50 in the US are offered in Table 2. These strategies offer actionable, practice- and policy-relevant recommendations for stakeholders in the US to advance early onset cancer screening and may be customizable for other global communities facing similar challenges in screening for early onset cancer (Table 2).

**Table 2:** Strategies across the socioecological model to enhance guideline-based early onset cancer screening.

Strategies to enhance risk-based cancer screening in adults under age 50		
Level	Strategy	
Individual	1. Develop health education and health communication campaigns to raise individual awareness of early onset cancer risk, cancer screening guidelines, and their family history of cancer. 2. Empower patients with the knowledge and practical tools to assist with making informed decisions and supporting conversation with family and providers. • Tier supplemental support via text, email, or other preferred outreach methods 3. Develop culturally-tailored materials and campaigns in multiple languages • Incorporate approaches that consider critical issues such as fear and/or mistrust of screening, perceived risk, health & digital literacy challenges, and other individual barriers to screening. 4. Develop and share digital tools tailored to individual needs, health literacy, and cultural identities that support knowledge transfer, behavioral activation, shared decision making, and sustained health behaviors for personalized, risk-based cancer screening	
Interpersonal	1. Build toolkits to assist individuals with family discussions about history of cancer and other pertinent medical history 2. Build toolkits to assist individuals in building relationships with their primary healthcare providers • Support individuals with bringing new or concerning symptoms to their provider, and initiating or participating in discussions about their family history of cancer and other pertinent family medical history 3. Develop and provide materials that are tailored to shared family and cultural norms	
Institutional	1. Identify and engage key stakeholders across clinical specialties and roles on early onset cancer screening initiatives • Medical specialties: primary care, internal medicine, gynecology/obstetrics, urgent care, medical oncology, surgical oncology, radiology, cancer genetics & high-risk specialists, and other subspecialties • Institutional stakeholders: physicians, mid-level providers, nurses, genetic counselors, administrators, researchers, and implementation specialists 2. Develop collaborative programs focused on risk-based screening in adults under 50 • Consider: risk assessment guidelines, structured family history collection as standard of care (with planned updates), patient identification & eligibility protocols, screening modalities & escalation procedures, and genetic testing guidelines 3. Develop institutional and health system wide screening policies and procedures consistent with up-to-date evidence and national screening guidelines 4. Develop and implement system-driven digital tools for clinical guidelines and decision support, including electronic medical record pathways and best practice alerts to standardize evidence-based risk assessment, screening recommendations, and referrals protocols. 5. Develop and implement cancer screening and cancer care navigation programs to assist in supporting patients with accessing routine screening and any necessary transitions to follow-up testing and/or treatment. • Automate outreach where possible to ensure universal and comprehensive connection with patients. • Identify financial support, non-profit grants, or other initiatives to offset high costs of screening and/or follow up care without insurance coverage 6. Develop and implement continuous clinical education on up-to-date risk-based cancer screening and genetic testing guidelines for adults under age 50 recommended by national organizations (e.g., NCCN, ACS, ASCO, FORCE). Mandate where possible. • Include continuous education on emerging trends in early onset cancer such as etiology, treatment, outcomes, etc. 7. Prioritize and support mixed methods research on early onset cancers (*see research priorities below*)	
Community	1. Cultivate and maintain relationships with community groups and local advocacy organizations to raise awareness about early onset cancer, cancer screening, and family cancer history. 2. Develop forums in which healthcare providers, public health experts, patient advocates, and local government officials can engage in active dialogue to advance screening for early onset cancers within communities. 3. Partner with local organizations to assist the community with reducing barriers to accessing cancer screening services and any required follow-up. • Potential partnerships: transportation assistance, mobile screening events (e.g., mammography vans), community health clinics, and community health workers	
Policy	1. Build and support active partnerships between healthcare providers and national organizations that set guidelines for cancer risk assessment and genetic testing (e.g., NCCN, ACS, ASCO, FORCE) 2. Encourage healthcare providers and administrators to engage local, state, and national government representatives to support payor coverage for high-risk cancer screening, cancer genetic testing, and abnormal finding follow-up care. 3. Partner with national organizations and grassroots initiatives to advocate for national policy change to support standardized access to risk-based cancer screening and follow-up care for adults under age 50	
Societal	1. Support the implementation of national-level guidelines for risk-based cancer screening in adult under age 50 to be implemented in all federally qualified health centers or health systems receiving federal support 2. Partner with public health agencies to develop and launch large scale public health campaigns to raise awareness on early onset cancer, risk-based cancer screening, genetic testing, and quality of life 3. Cultivate and maintain relationships with large national organizations and national advocacy groups to raise awareness about early onset cancer, cancer screening, and family cancer history. 4. Prioritize and fund mixed methods research on early onset cancers (*see research priorities below*)	
Research priorities		
Research domain	Research priorities	
Etiology & treatment	1. Identify cause(s) for early onset cancers 2. Distinguish between modifiable vs. non-modifiable risk factors 3. Evaluate optimal precision treatment protocols 4. Identify factors associated with aggressive disease, poor prognosis, and poor quality of life.	
Implementation	1. Explore barriers and facilitators of health system vs. community-based cancer screening initiatives and access pathways 2. Evaluate early onset cancer initiatives at the community, healthcare system, and national level	
Health behaviors	1. Explore digital health tool use to increase knowledge of cancer risk factors and activation of screening behavior for early onset cancers through pragmatic studies and randomized controlled trials where appropriate 2. Assess efficiency and efficacy of population-level initiatives to increase awareness and behavioral activation of risk assessment and genetic testing through cohort studies 3. Assess efficacy and acceptability of culturally-tailored educational resources through qualitative studies, pragmatic trials, and randomized controlled trials where appropriate 4. Explore health behaviors through cohort studies at the community, healthcare system, and national levels 5. Explore communication preferences and efficacy of behavioral activation in adults under age 50, including communication mode, channel, message, context, sender, and other critical factors	
Key recommendations		
Time frame	Stakeholder	Recommendation
Immediate to short-term	Individuals	1. Know your family cancer history 2. Share your family cancer history with your doctor 3. Consider genetic testing if appropriate	
	Healthcare providers	1. Discuss individual and family cancer history with all patients 2. Discuss appropriate risk assessments and genetic testing when indicated 3. Develop personalized, risk-based cancer screening plan for adults under age 50 based on established US guidelines	
Medium to long-term	Researchers	1. Advance research on etiology, implementation, health behaviors, and health communication related to early onset cancers (*see research priorities above*)	
	Policymakers, advocates, & health professionals	1. Adapt national guidelines to incorporate new etiological research findings 2. Leverage emerging scientific evidence to advance payor coverage of risk-based cancer screening and genetic testing in adults under age 50 3. Develop multilingual and culturally-tailored resources for individuals and providers based on emerging health behavior and health communication research	

NCCN, National Comprehensive Cancer Network; ACS, American Cancer Society; ASCO, American Society of Clinical Oncology; FORCE, Facing Hereditary Cancer Empowered.

Improving PRBS in younger adults requires expanding public awareness and empowering individuals to take action in collaboration with their healthcare providers (Table 2). This effort is currently hindered by challenges related to complex family and cultural dynamics, diverse family histories, limited family health discussions, and a complex health information landscape. Effective public communication and awareness campaigns must focus on empowering families to discuss cancer histories, support enhanced literacy on cancer risk and tailored screening, and provide accessible pathways to action. More resources like *My Family Health Portrait* are needed to guide family conversations about risk, genetic testing, and early screening.

Healthcare systems also need to help build support for early cancer detection among younger adults (Table 2) (19). Implementation of PRBS is an important consideration in clinical care. Given that cancer is most often a disease of aging, concerning symptoms in younger individuals may be minimized or overlooked, significantly delaying diagnosis (58). In addition, screening guidelines can be complex (23, 28, 29); therefore, continuing education for primary care providers and general practitioners on EOC risk is critical. Key elements for providers to consider include identifying individuals at high risk for specific cancers, following established cancer screening guidelines, and referring high-risk individuals to specialists as needed for further evaluation or management (33, 59, 60).

Furthermore, providers require efficient ways to obtain detailed family cancer histories from all patients to tailor cancer risk discussions and cancer screening recommendations, particularly for those individuals who are below the standard age-based screening guidelines (Table 2). Family history collection tools facilitate intake and organize information to identify patterns of high-risk cancer in families (61, 62). Validated risk assessment tools, such as Tyrer-Cuzick (IBIS) and BOADICEA (CanRisk), can provide estimates of lifetime risk of breast cancer based on family history and personal risk factors. If the lifetime risk for breast cancer is over 20% per these models, current NCCN guidelines recommend starting breast cancer screening no later than age 40 or 10 years prior to the youngest breast cancer diagnosis in a family, along with the use of breast MRI in the screening strategy (63–65). Clinical pathways that leverage the electronic medical record or use web-based or artificial intelligence-based approaches have also been promising in healthcare practices for mobilizing guidelines for patient care use (66, 67).

Multi-cancer early detection (MCED) assays represent a promising approach for blood-based cancer screening; however, they are not currently recommended for routine clinical use due to the absence of randomized evidence demonstrating a reduction in cancer-specific mortality, variable sensitivity for early-stage disease, and uncertainties regarding downstream diagnostic pathways and cost-effectiveness (68–72). As such, US national professional organizations such as NCCN do not include MCED testing in their guidance for cancer screening; the American Cancer Society states that clinicians do not need to initiate discussions of MCED testing with their patients until more data on clinical utility are available (68). Polygenic risk scores (PRS) also represent a promising approach to refining cancer risk stratification by integrating the cumulative association of common genetic variants for cancer risk (73); however, they are not currently recommended as stand-alone tools for routine cancer screening. Prospective studies demonstrate the feasibility of PRS-informed risk stratification; key limitations include their primary association with cancer incidence rather than mortality, need for longitudinal data on risk of cancer over time, applicability across diverse racial/ethnic populations, and the need for integration into multivariable risk models, which are the current standard of care (74–76). National organizations in the US, such as NCCN and ACS, do not currently endorse PRS as a stand-alone cancer risk stratification test. Clinical trial enrollment is encouraged to gain insights into how best to consider MCED and PRS testing for cancer screening or risk assessment in the future.

Continuing medical education on EOCs and personalized screening is necessary but not sufficient to achieve the desired EOC early-detection outcomes. Strong collaboration among primary care, oncology clinicians, cancer control specialists, genetic counseling programs, and other subspecialties is vital to implementing patient-centered cancer screening and management for younger individuals (Table 2) (77–79). Professional collaboratives, strong partnerships, and goal alignment are needed to dismantle the specialty care silos and support patients throughout their healthcare journey. Standardizing these practices in primary care visits and across specialty care may also empower patients and facilitate critical family conversations.

Many younger adults do not receive recommended early screening due to a range of access issues, including a lack of insurance coverage for screening before the standard recommended ages (19, 21). Even with access to screening, many may still face inadequate coverage for follow-up diagnostics, required monitoring, or treatment. These barriers are further amplified for those who experience other sociocultural challenges, such as limited financial stability, employment challenges, or differences in language and cultural norms (see Figure 1) (80). Without accessible and navigable pathways to screening, younger adults will continue to delay potentially life-saving screenings, monitoring, and treatment. Efforts to eliminate barriers and increase motivation for participation in screening are needed (Table 2) (80). Patient navigation programs can facilitate a tailored support pipeline from screening through diagnostics, monitoring, and follow-up care, ensuring patients have access to the entire care continuum (81–83). Navigators can also enhance health literacy, psychosocial support, and care coordination with the oncology team throughout this process, promoting continued adherence to recommended guidelines. Broad-scale health policy support for standardized access to and insurance coverage of tailored cancer screening, follow-up care, and navigation efforts for those at high risk of EOC could facilitate access for younger adults and improve outcomes.

While clear guidelines for tailored cancer screening protocols for high-risk individuals exist, there is a significant opportunity to expand understanding of EOCs. Research is needed to identify the causes of early onset cancers and factors impacting aggressive disease progression and poor outcomes. This knowledge development is key to refining tailored cancer screening recommendations (Table 2). Research is also needed to discern how younger audiences interact with the healthcare system. There is a broad knowledge gap in best practices for sharing cancer risk information with younger adults, which encompasses understanding information-seeking habits, behavioral motivations, trusted messengers, digital health trends, and social media influence. Collaborating with young adult stakeholders in campaign design will be critical for delivering content that resonates and drives desired actions (e.g., family cancer history discussions, provider discussions, genetic testing, and/or tailored cancer screening). Cross-disciplinary partnerships to advance these efforts would ideally include experts in public health, social work, genetics, oncology, communications, psychology, and the young adult community.

Given the rise in early onset cancers, a compelling need exists for enhanced awareness, public empowerment, patient-centered healthcare and policy systems, and innovative research. With continued research, digital and social innovation, diverse stakeholder collaboration, and public campaigns targeting younger adults, there is significant potential to more effectively address the needs of this growing population.

## Data Availability

The original contributions presented in the study are included in the article/supplementary material, further inquiries can be directed to the corresponding author.
